# Risk Stratification in Lung Resection

**DOI:** 10.1007/s40137-016-0158-x

**Published:** 2016-09-20

**Authors:** Michele Salati, Alessandro Brunelli

**Affiliations:** 1 Division of Thoracic Surgery, Ospedali Riuniti Ancona, Via Conca 1, 60020 Ancona, Italy; 2 Department Thoracic Surgery, St. James’s University Hospital, Beckett Street, Leeds, LS9 7TF UK

**Keywords:** Preoperative evaluation, Cardiac risk, Co-morbidities, Pulmonary function, Exercise test, Operative Risk, Morbidity, Mortality, Lung resection, Lung cancer surgery

## Abstract

**Purpose of Review:**

Surgery is considered the best treatment option for patients with early stage lung cancer. Nevertheless, lung resection may cause a variable functional impairment that could influence the whole cardio-respiratory system with potential life-threatening complications. The aim of the present study is to review the most relevant evidences about the evaluation of surgical risk before lung resection, in order to define a practical approach for the preoperative functional assessment in lung cancer patients.

**Recent Findings:**

The first step in the preoperative functional evaluation of a lung resection candidate is a cardiac risk assessment. The predicted postoperative values of forced expiratory volume in one second and carbon monoxide lung diffusion capacity should be estimated next. If both values are greater than 60 % of the predicted values, the patients are regarded to be at low surgical risk. If either or both of them result in values lower than 60 %, then a cardiopulmonary exercise test is recommended. Patients with VO2max >20 mL/kg/min are regarded to be at low risk, while those with VO2max <10 mL/kg/min at high risk. Values of VO2max between 10 and 20 mL/kg/min require further risk stratification by the VE/VCO2 slope. A VE/VCO2 <35 indicates an intermediate-low risk, while values above 35 an intermediate-high risk.

**Summary:**

The recent scientific evidence confirms that the cardiologic evaluation, the pulmonary function test with DLCO measurement, and the cardiopulmonary exercise test are the cornerstones of the preoperative functional evaluation before lung resection. We present a simplified functional algorithm for the surgical risk stratification in lung resection candidates.

## Introduction

During the last 40 years, an increasing amount of papers addressed the topic of perioperative risk assessment in the field of lung surgery.

At the end of the eighties, the attention was focused on the spirometric parameters, particularly lung volumes and flows, as potential predictors of poor outcome when preoperatively impaired. Nevertheless, as it became clear in the following decade, the spirometric evaluation was not able to discriminate per se the surgical risk, and some other factors, such as the lung diffusion capacity, had to be considered for predicting the risk of morbidity and mortality. These parameters were adopted as the standard of the preoperative functional assessment before lung resection at the end of the last century. Moreover, they were considered the first-level examination step before proceeding to more sophisticated evaluation strategies, as reported in the most recent algorithms for the preoperative fitness assessment. As a consequence, during the last 15 years, a growing evidence has highlighted the role of the ergometric capacity assessment tested through the cardiopulmonary exercise test as the ultimate evaluation tool in order to define the surgical risk.

The present study offers an overview of the most relevant papers about the risk stratification before lung resection with special attention to their clinical relapses. At the end of each paragraph, some practical recommendations are summarized that were condensed in order to propose a functional evaluation algorithm useful for daily clinical practice.

This article does not contain any studies with human or animal subjects performed by any of the authors.

## Cardiologic Evaluation

As recommended by the most recent algorithms concerning the functional evaluation of candidates to lung resection, the first step for estimating the surgical risk is represented by an accurate cardiac evaluation [1••, 2].

In fact, this should be the preliminary patient assessment, before proceeding with the pulmonary and ergometric evaluation, since the presence of unstable cardiac disease could per se influence an increased surgical risk. As a consequence, an optimization of the cardiac function by medical or surgical therapy is strongly recommended in these patients before proceeding with the planned lung resection.

In order to identify the category of patients with a higher chance of postoperative cardiac adverse events due to a pre-existent cardiac disease or some other pathologic factors, in 1999 Lee et al. developed the Revised Cardiac Risk Index (RCRI) for stable patients undergoing non-urgent major non-cardiac surgery [3]. This risk stratification tool was refined in 2010 by Brunelli et al. proposing a new risk score (ThRCRI) derived from an homogeneous population of 1696 patients submitted exclusively to major lung resection (1426 pulmonary lobectomies and 270 pneumonectomies) [4]. In order to calculate the ThRCRI of a lung resection candidate, four different factors (each of them having a specific weight for the final index) should be taken into account:

- 1. History of coronary artery disease, 1.5 points.
- 2. Cerebrovascular disease, 1.5 points.
- 3. Serum creatinine level greater than 2 mg/dl, 1 point.
- 4. Pneumonectomy, 1.5 points.

Summing the points of each factor, the patient’s aggregate ThRCRI is obtained, which ranges from a minimum of 0 to a maximum of 5.5. This value identifies four different risk classes predicting an incremental risk of cardiac morbidity:

- Class A: 0 points. Risk of cardiac complication: 1.5 %.
- Class B: 1–1.5 points. Risk of cardiac complication: 5.8 %.
- Class C: 2–2.5 points. Risk of cardiac complication: 19 %.
- Class D: >2.5 points. Risk of cardiac complication: 23 %.

In 2011, the ThRCRI was validated in two external populations of 2621 and 1255 patients [5•, 6]. Both studies verified that patients with a ThRCRI greater than 2.5 have a risk of major cardiac complications, ranging from 13 to 18 %, confirming the reliability and usefulness of the score.

Following these results, the most recent guidelines of the ACCP about the physiologic evaluation of patients considered for resectional surgery [1••] suggested that those ones with a ThRCRI ≥2 should be referred for a formal cardiology evaluation and eventually to tests and treatments as recommended by the American Heart Association and the American College of Cardiology guidelines [7].

### Suggestions

- The cardiac evaluation is the first preliminary step of the patient’s functional status
- Calculate the ThRCRI for each lung resection candidate
- In case of a ThRCRI <2, proceed with the pulmonary functional evaluation
- In case of a ThRCRI ≥2, optimize the cardiac function before considering lung surgery

## Forced Expiratory Volume at First Second (FEV1) and Predicted Postoperative FEV1 (ppoFEV1)

The roles of the FEV1 and of its derived parameter ppoFEV1 in the functional assessment before lung resection have considerably changed during the last decade.

Since the eighties, several papers have been published addressing the importance of the FEV1 in defining the risk of morbidity and mortality for lung surgery. The most relevant ones are reported in the following list:

- 1988: Nakahara et al. Retrospective observational study. Cohort: 157 patients submitted to anatomic lung resection. The ppoFEV1 showed a correlation with the postoperative respiratory complications. In the group of patients with ppoFEV <30 %, the mortality rate was about 60 % [8].
- 1989: Markos et al. Retrospective observational study. Cohort: 47 patients submitted to lobectomy (29) and pneumonectomy (18). The ppoFEV1 was a predictor of complications and death. No patients with a ppoFEV1 >40 % died, while three of six patients with a ppoFEV1 <40 % died in the perioperative period [9].
- 2005: Magdeleinat et al. Retrospective observational study. Cohort: 106 patients submitted to lung resection (17 sublunar resections) with a preoperative FEV1 and/or FVC <50 %. The overall morbidity rate was 70 % and the mortality rate 8.5 %. 21 % of patients required prolonged mechanical ventilation (mean 11 days). The morbidity rate raised up to 100 % for patients with a ppoFEV1 loss >15 % [10].
- 2006: Licker et al. Retrospective observational study. Cohort: 1239 consecutive thoracotomies. The FEV1 <60 % was an independent risk factor of respiratory complications, including prolonged air leak (OR = 2.7) and 30-day mortality (OR = 1.9) [11].
- 2008: Ferguson et al. Retrospective observational study. Cohort: 1046 patients submitted to major lung resection. Using a classification and regression tree analysis, FEV1 turned out to be an independent predictor of pulmonary morbidity and cardiovascular complications. The FEV1 was not related to mortality [12].
- 2010: Berry et al. Retrospective observational study. Cohort: 340 patients submitted to open or video-assisted lobectomy and with a FEV1 or a DLCO <60 %. The overall morbidity rate was 48 % and the mortality rate 5 %. Within the thoracotomy patients, the level of FEV1 was inversely correlated with the pulmonary complication rate. The FEV1 was an independent predictor of respiratory morbidity for the open patients but not for the ones treated with a thoracoscopic approach [13].

Most recently, growing evidence has questioned the role of the FEV1 in defining the risk before the surgical treatment. In fact, several studies showed that the FEV1 failed to estimate the postoperative outcome in some categories of patients (such as the ones with an higher COPD grade). At the same time, some Authors demonstrated the limits of the ppoFEV1 in predicting the postoperative pulmonary function, especially in the early postoperative period.

- 1998: Korst. Retrospective observational study. Cohort: 32 patients submitted to lobectomy. The COPD index is inversely correlated with the residual FEV1 measured after the operation (follow-up between 4 months and 2 years). Patients with a FEV1 <60 % and a FEV1/FVC <0.6 experienced an increase of the FEV1 after lobectomy (mean FEV1 increase: 3.7 %) [14].
- 1999: Carretta et al. Retrospective observational study. Cohort: 35 patients submitted to lobectomy. Patients with an higher grade of emphysema had stable or slight improvement of the FEV1 and FVC values after the lobectomy (mean time of follow-up pulmonary function assessment: 4.7 months). In this group of patients, the postoperative FEV1 increases of about 6 % in comparison to the preoperative value [15].
- 2001: Santambrogio et al. Retrospective observational study. Cohort: 88 patients submitted to lobectomy. Patients encountering the spirometric criteria of COPD (FEV1 <80 %) showed a lesser reduction of the FEV1 6 months after the operation in comparison to the ones with normal pulmonary function. The postoperative FEV1 decrease was −3.2 % for the COPD group and −14.9 % for the non-COPD group (*p* < 0.001) [16].
- 2002: Brunelli et al. Retrospective observational study. Cohort: 544 patients submitted to lobectomy (441) or pneumonectomy (130). The postoperative complications rate (overall morbidity rate: 21.1 %, overall mortality rate: 2.9 %) did not differ between the patients with a preoperative FEV1 >70 % (group A: 450 pts) and the ones with a FEV1 <70 % (group B:93 pts). The predictors of complications within group A were FEV1, ppoFEV1, and COPD index. No spirometric predictors of outcome were identified for the group B [17].
- 2003: Sekine et al. Retrospective observational study. Cohort: 521 patients submitted to lobectomy. The postoperative FEV1 measured 1 month after the operation showed a decrease of 13.1 % compared to the preoperative values within the group of COPD patients (FEV1 <70 % and FEV1/FVC <0.7, 48 pts), while the reduction for the non-COPD patients was 29.2 % (*p* < 0.001). The measured postoperative FEV1/ppoFEV1 ratio was grater than 1 for the COPD patients [18].
- 2007: Brunelli et al. Retrospective observational study. Cohort: 200 patients submitted to lobectomy (180 pts) and pneumonectomy (20 pts). Within the lobectomy patients, the actual postoperative FEV1 measured at discharge, 1 and 3 months after the operation, was −11 %, similar, and +6 % in comparison to the calculated ppoFEV1. The actual postoperative FEV1 overestimated the ppoFEV1 especially for the patients with lower expected FEV1 after the operation [19].
- 2005: Brunelli et al. Prospective observational study. Cohort: 190 patients submitted to lobectomy (161 pts) and pneumonectomy (29 pts). The authors presented a regression equation in order to optimize the calculation of the ppoFEV1 taking into account multiple correction parameters. The estimated percentage of FEV1 reduction was obtained by the formula: [21.34–(0.47 × age) + (0.49 × percentage of functioning parenchyma removed during operation) + (17.91 × COPD index)] [20].

Taking into account the reported evidences, the FEV1 has progressively lost the role of defining independently from other parameters the functional status and, as a consequence, the risk before major lung resection.

In fact, the most recent guidelines, developed for managing the preoperative physiologic evaluation of the patients who were candidates to lung surgery, considered the FEV1 as one of the factors that can lead the evaluation algorithm rather than the single functional variable that was able to select patients for surgical treatment [1••, 2–4, 5•, 6–21].

### Suggestions

- A formal spirometry with FEV1 measurement should be performed for each patient who was a candidate to lung resection.
- In case of a ppoFEV1 <60 %, the patient should be considered at an higher operative risk.
- In case of a ppoFEV1 <60 %, the patient should be evaluated with a second level functional test as a formal cardiopulmonary exercise test.
- Do not exclude from the operation any patient solely on the basis of a low ppoFEV1 value.

## Carbon Monoxide Lung Diffusion Capacity (DLCO)

Evidences highlighting the DLCO as an additional and independent lung function parameter that was able to define the surgical risk in pulmonary resection were first published by Ferguson et al. about 25 years ago. In the first paper, these Authors showed the correlation between an impaired DLCO and the development of postoperative respiratory complications and death. In particular, analyzing 237 patients submitted to major lung resection (73 pneumonectomies), they found a complication and mortality rates of 40 and 20 %, respectively, in those patients with a DLCO <60 % [22]. In 2010, Berry obtained similar results in a retrospective study on 167 patients submitted to open lobectomy. The logistic regression confirmed that the DLCO was associated to pulmonary complications, which reached the rate of about 40 % in those patients with a DLCO <45 % [23].

Moreover, an even stronger ability to relate with the postoperative outcome was then demonstrated for the derived ppoDLCO, again by Ferguson in a study on 376 patients (246 lobectomies, 38 bilobectomies, 92 pneumonectomies). The ppoDLCO and age turned out to be the only predictors of any type of complications and mortality among 23 physiologic and spirometric preoperative parameters [24]. These findings were confirmed most recently by other studies [25, 26].

Finally, several papers documented that the DLCO maintains its role as risk factor before lung resection independently from the COPD status of the patients. In a multi-centric study on 872 patients submitted to lung resections (129 wedges/segmentectomies, 611 lobectomies/bilobectomies, 132 pneumonectomies), Brunelli demonstrated that age and ppoDLCO <40 % were the only predictors of morbidity in the group of patients without an airflow limitation (FEV1 >80 %: 508 patients, morbidity rate for its with ppoDLCO >40:17.5 % vs morbidity rate for its with ppoDLCO <40:37 %, *p*: 0.004). Moreover, showing a low correlation coefficient between FEV1 and DLCO for the entire population as well as for subgroups of analysis, the Authors recommended the DLCO measurement before lung surgery for all the patients, irrespectively of the FEV1 values [27].

The central role of the ppoDLCO for the risk stratification was corroborated by a subsequent analysis of Ferguson on 1008 patients submitted to anatomic major lung resection. Dividing the population into two groups (450 COPD patients and 558 non-COPD patients, COPD was defined as FEV1/FVC <0.7), the multivariate analysis showed that the ppoDLCO was a significant predictor of pulmonary complications and mortality both in patients with and without COPD. The Authors also documented a linear increase of pulmonary complications and mortality with a progressive education of the ppoDLCO values similar for the two groups of patients [28].

### Suggestions

- A systematic DLCO measurement should be performed for each patient who was a candidate to lung resection irrespectively of the FEV1 value registered.
- In contrast with the FEV1, the DLCO maintains its ability in evaluating the risk of complications independently from the COPD status of the patients.
- In case of a ppoDLCO <60 %, the patient should be evaluated with a second level functional test as a formal cardiopulmonary exercise test.
- Do not exclude from the operation any patient solely on the basis of a low ppoDLCO value.

## Cardiopulmonary Exercise Testing (CPET)

Considering the most recent guidelines for the physiologic evaluation before lung surgery, the formal high tech cardiopulmonary exercise test (CPET) is considered the gold standard for the functional assessment and the risk stratification of candidates to pulmonary resection [1••].

The first evidences addressing the role of CPET in assessing the surgical risk were published during the nineties. The exercise capacity expressed as percentage of the predicted value of the maximum oxygen consumption (VO2max  %) was the first ergometric parameter found to be associated with postoperative complication and mortality. Bolliger et al. analyzed 80 patients submitted to lung resection (14 minor resections) and evaluated by a symptom-limited CPET. The VO2max  % turned out to be the best predictor of complication at the regression analysis. Patients with a VO2max  % <60 % had a high risk of postoperative adverse events up to 89 % [29]. These data were confirmed by a prospective trial performed from 1990 to 1997 on 125 anatomic lung resections. Among 19 demographic, spirometric, surgical, and ergonometric parameters, the only parameters associated with postoperative complications were the extent of resection and the VO2max  %. Moreover, the Authors estimated the risk of complications at different levels of VO2max  % for each type of resection performed. In particular, in case of a VO2max  % = 60 %, they found a probability of complication varying from 45 % in case of segmentectomy, to 78 % in case of pneumonectomy [30]. In 2005, Win et al. corroborated these findings and stated that a VO2max  % threshold between 50 and 60 % should be considered the limit, above which resections should be performed with a low risk of complications and mortality [31].

Nevertheless, most recent papers reconsidered the importance of the VO2max  %, demonstrating that the absolute value of the maximal oxygen consumption measured in ml/kg/min (VO2max) was the optimal ergometric parameter in order to quantify the risk for major lung resections. Some of the most relevant studies are reported as follows:

- 2007: Loewen et al. Prospective multi-institutional observational study. Cohort: 346 patients submitted to thoracotomy wither without lung resection (73 sublobar resections, 7 exploratory thoracotomy). The Authors found that patients at risk for postoperative complications and high mortality rate were the ones with a VO2max <15 ml/kg/min [32].
- 2007: Bayram et al. Prospective multi-institutional observational study. Cohort: 55 patients submitted to major lung resection. The Authors did not observe any adverse events in patients with a VO2max >15 ml/kg/min. The 28 patients with a VO2max < 15 ml/kg/min experienced a postoperative complication rate of 39 % (2 patients died) [33].
- 2009: Brunelli et al. Retrospective observational study. Cohort: 204 patients submitted to major lung resection (177 lobectomies, 24 pneumonectomies). The VO2max turned out to be the best predictor of respiratory complications. Patients with a VO2max <12 ml/kg/min had a mortality rate of 13 %, while no mortality was observed in patients with a VO2max >20 ml/kg/min. Finally, the Authors showed that, at the ROC analysis, the best threshold for predicting both pulmonary complication and death was a VO2max <12 ml/kg/min [34].
- 2011: Licker et al. Retrospective observational study. Cohort: 210 patients with FEV1 <80 % submitted to lung resection. The VO2max was a predictor of cardiopulmonary complication and death at the multivariate analysis including preoperative clinical, surgical, and ergometric variables. Patients with a VO2max <10 ml/kg/min had a risk of total morbidity, cardiovascular morbidity, and cardiac morbidity of 65, 39, and 35 %, respectively, in case of major resection [35].

Based on these evidences, the VO2max obtained at the CPET is considered by the recent functional algorithm as the definite and most reliable parameter stratifying the risk. Using the VO2max value as an indicator of the global performance status of the patients, it can be decided the best treatment option for lung resection candidates.

### Suggestions

- The high tech CPET with the VO2max measurement is the most reliable parameter for defining the surgical risk in lung resection candidates.
- Perform a formal CPET in any patient with an impaired ppoFEV1 and ppoDLCO.
- In case of a VO2max >10 ml/kg/min, the risk for a major lung resection is acceptable varying from moderate to low.
- In case of a VO2max <10 ml/kg/min, the risk for a major lung resection is high, and the patient should be considered for minor resection or alternative non-surgical therapies.

## Minute Ventilation to Carbon Dioxide Output (VE/VCO2) Slope

Recently, several papers have been published in order to verify if ergometric parameters other than the VO2max have the potential for predicting the postoperative surgical outcome, and consequently could be used as risk stratification factors in patients submitted to lung resection [36–40].

The most promising parameter is represented by the slope of the minute ventilation to carbon dioxide output ratio (VE/VCO2). This relationship, elsewhere reported as ventilatory efficiency curve, describes the potential of the cardio-respiratory system in increasing the CO2 output through a higher minute ventilation during the exercise. An abnormal rise of the VE/VCO2 slope values could be related both to pulmonary and cardiac diseases, such as COPD, pulmonary hypertension, or heart failure [41, 42].

In 2010, Torchio et al. published a retrospective study on 145 COPD patients submitted to major lung resection (including 39 pneumonectomies) and evaluated them by a formal preoperative CPET. The mortality and cardiopulmonary morbidity rates were 3.4 and 14.5 %, respectively. The VO2max turned out to be the best predictor of morbidity after the logistic regression, while the only parameter associated with mortality was the VE/VCO2 slope. In particular, a VE/VCO2 slope ≥34 was related to a risk of mortality of 5.5 %. Therefore, the Authors recommended the screening of major lung resection candidates for potential ventilatory insufficiency to refine the risk of mortality, irrespectively of the VO2max value reached at the preoperative CPET.

Two years later, Brunelli et al. analyzed a cohort of 225 patients submitted to lobectomy (197) and pneumonectomy (28) after a complete functional evaluation including a CPET independently from the preoperative or ppo FEV1 and DLCO values. The cardiopulmonary morbidity rate was 23 %, while a total of 25 patients (11 %) experienced a postoperative pulmonary adverse event. This group of patients registered a VE/VCO2 slope significantly higher in comparison to the uncomplicated patients (34.8 vs 30.9, *p* 0.001). Moreover, the Authors found that, after logistic regression analysis the VE/VCO2 slope remained the only predictor of respiratory complications, and those patients with a VE/VCO2 slope ≥35 had a 3-fold higher probability of experiencing respiratory complications during the postoperative period.

The role of the VE/VCO2 slope as an outcome predictor after lung resection was further confirmed by Shafiek and coll. [43•]. In a retrospective study on 83 COPD patients, the Authors verified that a VE/VCO2 slope >35 was the stronger predictor of mortality and morbidity, even if tested in association with the VO2max.

These evidences support the use of VE/VCO2 slope as a relevant parameter for defining the preoperative risk before lung surgery. Hopefully, further studies could strengthen the role of this risk factor in order to include it within functional evaluation algorithms.

## Conclusions

Based on the evidences from the literature reported above and the personal experience of the authors, we propose a simplified functional algorithm.

**Fig. 1:**
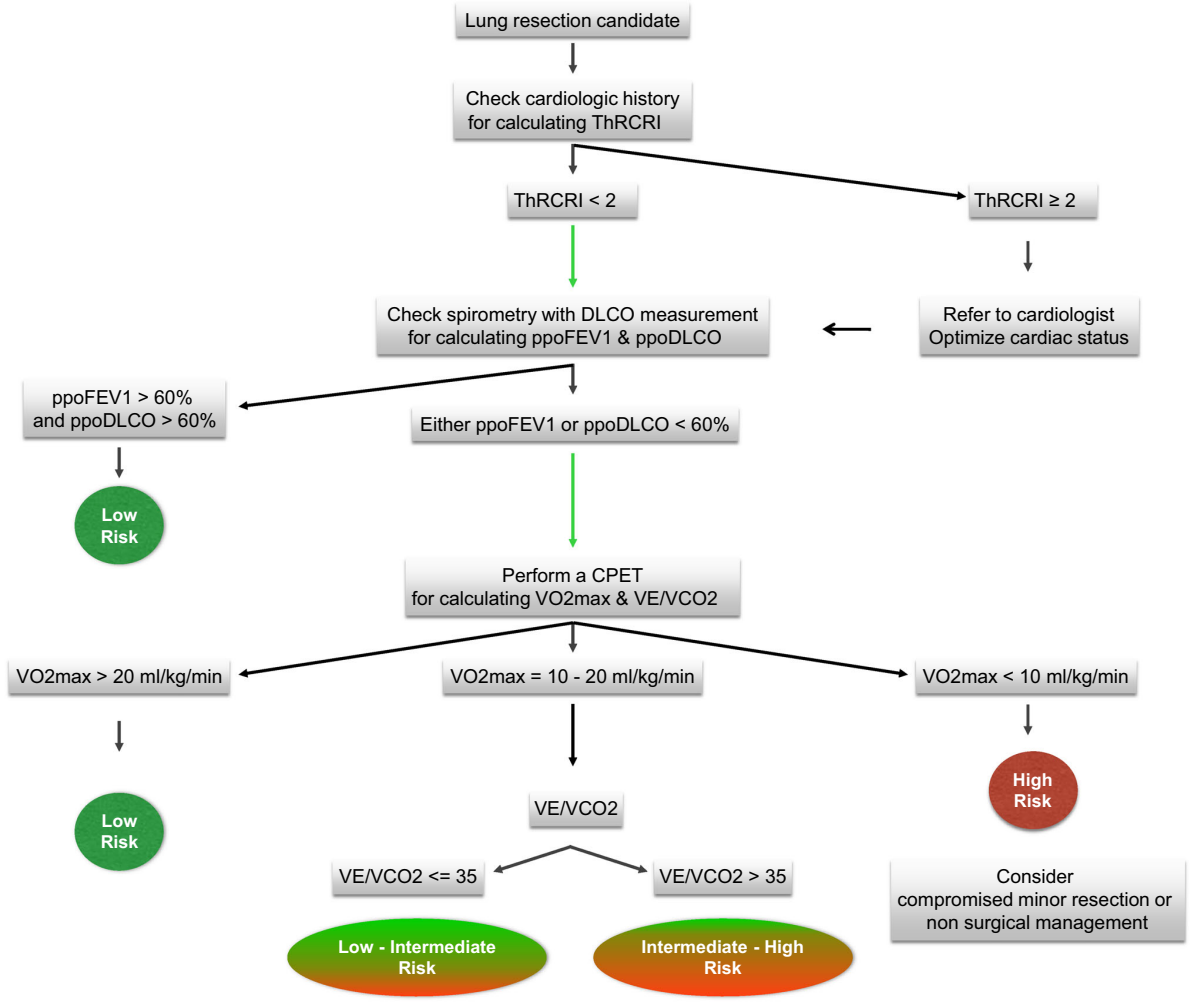
Simplified functional algorithm based on the reported evidences and authors experience (see Conclusions for explanation)

As shown in Fig. 1, the flow chart starts from a cardiac evaluation based on the estimation of the ThRCRI. If the patient has a ThRCRI greater than 2, then a specialist referral to a cardiologist is recommended to optimize their cardiac status.

Following cardiac risk evaluation, a pulmonary function test is obtained with measurement of both FEV1 and DLCO, and split lung function is calculated based on the planned extent of the resection.

If both ppoDLCO and ppoFEV1 are greater than 60 % of predicted values, then no further tests are advised as they would be regarded as low risk patients.

If either or both of these parameters are lower than 60 %, then a cardiopulmonary exercise test is recommended.

The two parameters that should be taken into consideration are the maximum oxygen consumption (VO2max) and the efficiency slope (VE/VCO2). Patients with VO2max greater than 20 mL/kg/min are regarded as low risk for surgery. Those with VO2max lower than 10 mL/kg/min are deemed at high risk for anatomic lung resection. Patients with values of VO2max falling in between 10 and 20 mL/kg/min would be better risk stratified by the VE/VCO2 slope. If VE/VCO2 is lower than 35, then they can be considered at intermediate-low risk, while a value above 35 should be considered a marker of intermediate-high risk.
